# Trends in employment inequalities for people with disability, Australia 2003 to 2023

**DOI:** 10.1136/oemed-2026-110930

**Published:** 2026-07-29

**Authors:** Lu Ye, Glenda M Bishop, Zoe Aitken

**Affiliations:** 1 Melbourne School of Population and Global Health, University of Melbourne, Carlton, Victoria, Australia

**Keywords:** Epidemiology, Public health, Disabled Persons, Occupational Groups, Longitudinal studies

## Abstract

**Objectives:**

People with disability are less likely to participate in the labour force and be employed compared with people without disability. However, it is not known whether these inequalities have changed over time. We therefore assessed whether disability-related inequalities in employment have changed over time among adults aged 20–64 years in Australia.

**Methods:**

We analysed 21 years of data (2003–2023) from the Household, Income and Labour Dynamics in Australia Survey. Trends were investigated to describe changes in inequalities in employment, unemployment and not in the labour force (NILF) between people with and without disability. Analyses were further disaggregated for age group, sex and disability subgroups.

**Results:**

From 2003 to 2023, people with disabilities experienced persistent and substantial inequalities in employment outcomes. Neither absolute nor relative inequalities in employment outcomes changed and relative inequalities for NILF widened over time. People with disability were 30% to 40% less likely to be employed, 1.4 to 2.7 times more likely to be unemployed and 2.0 to 2.8 times more likely to be NILF compared with people without disability. The greatest relative inequalities in employment were experienced by older working-age people with disability, people with intellectual or learning disability, brain injury or stroke and psychological disability.

**Conclusions:**

This descriptive study adds to previous findings showing employment inequalities by demonstrating that disability-related inequalities in employment have been sustained for two decades in Australia without improvement. As a key social determinant of health, the findings are a critical public health concern.

**WHAT IS ALREADY KNOWN ON THIS TOPIC**

- People with disability are less likely to participate in the labour force, be employed and more likely to be unemployed than people without disability.
- There is no evidence on whether these inequalities have changed over time.

**WHAT THIS STUDY ADDS**

- Inequalities in employment between Australians with and without disability have persisted and not improved for 21 years, regardless of age, sex and type of disability.
- The greatest relative inequalities were experienced by older people with disability, people with intellectual or learning disability, brain injury or stroke and psychological disability.

**HOW THIS STUDY MIGHT AFFECT RESEARCH, PRACTICE OR POLICY**

- Government and employment service providers should strengthen accountability, monitoring and evaluation of disability employment policies and programmes to ensure they are effective in improving employment outcomes and reducing disability-related inequalities.

## Introduction

 Employment is a key social determinant of mental health that provides financial resources as well as latent psychological benefits including time structure, social connection, a sense of social purpose, status and regular activities.^1^ Systematic reviews have consistently demonstrated that employment is associated with lower risks of depression and psychological distress and improved mental health outcomes.^2 3^ Conversely, unemployment has detrimental effects on mental health due to the loss of the financial and latent benefits of work, contributing to social isolation^4^ and increasing risks of depression, anxiety, mental distress and reduced life satisfaction.^5 6^

People with disability represent a population group that experiences notable inequalities in employment,^7^ with their full and effective participation in society often hindered by the complex interplay between impairments, personal characteristics and environmental barriers.^8^ The United Nations reports that working-age adults with disability are less likely to be employed and more than twice as likely to be unemployed as people without disability across 27 Organisation for Economic Co-operation and Development (OECD) countries, including Australia.^9 10^ These large employment inequalities are particularly concerning given that approximately 16% of the world’s population and 21.4% of Australia’s population have disability, and people with disability experience poorer mental health than people without disability.^11 12^ The relationship between employment and mental health outcomes has been well documented among people with disability, as employment plays a critical protective role in their mental health, whereas unemployment is associated with poorer mental health outcomes.^13 14^

People with disability experience multifaceted and interacting barriers that reduce their likelihood of gaining and maintaining employment, including limited availability of suitable jobs, inadequate education and vocational training, persistent stigma and work discrimination, and inaccessible transport.^15–17^ The inequalities in employment are also likely to be driven by intersecting identities, such as age and sex/gender, as well as type of disability. Thus, examining how inequalities in employment vary across subgroups of people with disability is crucial for understanding which populations are most disadvantaged and for guiding targeted policy action.

The United Nations Convention on the Rights of Persons with Disabilities (UNCRPD) emphasises the rights of people with disability to work on an equal basis with others (Article 27).^8^ As a signatory, Australia has implemented a series of national policies over recent decades, including the National Disability Strategy 2010–2020,^18^ followed by Australia’s Disability Strategy 2021–2031,^19^ Employ My Ability – the national disability employment strategy introduced in 2021,^20^ and the National Disability Insurance Scheme (NDIS) implemented progressively from 2013.^21^ However, there is an existing knowledge gap as to whether these policy strategies reduced inequalities in employment for people with disability.

This descriptive study aimed to quantify employment inequalities among working-age adults with and without disability, and to determine whether these inequalities changed over time. It further examined how employment inequalities differed across age, sex and disability groups.

## Methods

### Data source

The data were drawn from the Household, Income and Labour Dynamics in Australia (HILDA) Survey, a nationally representative longitudinal study conducted annually since 2001. Information was collected from all household members aged 15 years and older using a combination of interviews and self-completed questionnaires. The original sample included 13 969 individuals from 7682 households (household response rate: 66%).^22^ This sample has been followed up in subsequent waves, with top-up samples added in 2011 and 2023 to maintain representativeness. Response rates among continuing participants have remained high, ranging from 86.9% to 97.0% across 23 waves.^23^ Our sample used data from people aged 20–64 years from 21 waves (2003–2023).

### Disability status

Disability status (with or without disability) was assessed by asking participants whether they had ‘any long-term health condition, impairment or disability […] that restricts you in everyday activities, and has lasted or is likely to last for 6 months or more’. Information on the type of disability was determined using showcards listing specific examples of impairments, disabilities and health conditions. As the showcards were updated in 2003 to include a broader range of conditions, only data from 2003 onwards were used to ensure consistency in the disability definition. The reported conditions were categorised into six disability groups: sensory or speech, physical, intellectual or learning, psychological, brain injury or stroke, and other disability/not specified. Participants could report multiple conditions and be classified into more than one disability group.

### Employment

Employment status was based on job-related activities in the previous 4 weeks and defined using three categories: (i) employed (engaged in any type of economic activities); (ii) unemployed (not in paid employment but actively seeking work and available to start); and (iii) not in the labour force (NILF).^24^ Participants were classified as NILF if they were neither employed nor actively looking for work, or if they were actively seeking work but not available to start.^25^

### Statistical analysis

The analytic sample included 240 591 observations from 26 932 participants aged 20–64 years, who responded to the disability question (99.6% of respondents). There were no missing data for employment status, age and sex. Population-weighted descriptive analysis was used to describe the demographic and socioeconomic characteristics (age group, sex, country of birth, remoteness, highest level of education and equivalised household income) of people with and without disability in the first and last waves contributing to the analysis (2003 and 2023).^26^ Survey weights were used to adjust for clustering, stratification, and non-response in the survey design, with standard errors calculated using the Taylor Series linearisation method.^27^

To describe the prevalence of employment, unemployment and NILF for people with and without disability in each wave, we calculated population-weighted age-standardised estimates of proportions (and 95% CIs) for each employment status, stratified by disability status. To adjust for differences in age structure between people with and without disability, we applied direct age standardisation using the age distribution of people with disability in Wave 23 of the survey as the reference population, grouped in 5 year age bands.^28^

Absolute and relative inequalities in employment status, and 95% CIs, between people with and without disability were then estimated for each wave. Absolute inequalities in employment, unemployment and NILF were calculated as the difference in prevalence between people with and without disability. Relative inequalities in employment, unemployment and NILF were calculated as the ratio of the prevalence for people with disability to the prevalence for people without disability. To assess differences between groups and over time, we examined each measure (prevalence, absolute and relative inequality) for non-overlapping 95% CIs. Analyses were repeated and stratified by age group, sex and disability group, with the latter restricted to analysis of employment and NILF due to small numbers of people unemployed when disaggregated by disability group.

All statistical analyses were performed using Stata/SE 18.0. Graphs were generated using R version 4.5.1, RStudio version 2025.09.1+401 and ggplot 2 V. 4.0.0.

## Results

### Population characteristics

The population-weighted prevalence of disability of people aged 20–64 years remained stable, with a prevalence of 23.8% in 2003 and 23.0% in 2023. Table 1 shows the population-weighted characteristics of participants with and without disability in the first and final waves. Among participants with disability, in 2003, the most common disability group was physical disability, followed by other/not specified, sensory or speech and psychological disability. In 2023, the most common group was other/not specified, followed by physical, psychological and sensory or speech disability. From 2003 to 2023, the proportion of people with physical disability declined (from 57.4% to 43.7%), while the proportions more than doubled for people with psychological disability (from 13.6% to 30.4%) and intellectual or learning disability (from 3.3% to 7.8%).

**Table 1:** Population-weighted estimates of demographic and socioeconomic characteristics for people with and without disability aged 20–64 years in 2003 and 2023

Characteristics	2003				2023			
	No disability		Has disability		No disability		Has disability	
	(n=7339)		(n=2298)		(n=8606)		(n=2733)	
	%	95% CI	%	95% CI	%	95% CI	%	95% CI
Disability groups*								
Sensory or speech			18.7	16.9 to 20.7			16.9	15.0 to 19.0
Physical			57.4	54.9 to 60.0			43.7	41.0 to 46.5
Intellectual or learning			3.3	2.3 to 4.6			7.8	6.5 to 9.2
Psychological			13.6	11.9 to 15.6			30.4	28.0 to 32.9
Brain injury or stroke			2.7	2.0 to 3.5			4.2	3.3 to 5.3
Other/not specified			49.7	47.1 to 52.2			58.0	55.2 to 60.7
Age groups								
20–34 years	39.4	37.7 to 41.1	21.6	19.6 to 23.8	38.3	36.6 to 40.0	29.0	26.6 to 31.5
35–49 years	38.4	36.8 to 39.9	35.3	32.9 to 37.8	35.5	33.7 to 37.5	26.9	24.5 to 29.4
50–64 years	22.3	20.8 to 23.8	43.1	40.4 to 45.8	26.2	24.8 to 27.6	44.1	41.3 to 47.0
Sex								
Male	48.8	47.7 to 49.9	52.9	50.9 to 54.9	50.8	49.6 to 52.0	45.5	43.1 to 48.0
Female	51.2	50.1 to 52.3	47.1	45.1 to 49.1	49.2	48.0 to 50.4	54.5	52.0 to 56.9
Country of birth								
Australia	71.6	69.1 to 73.9	72.3	69.3 to 75.1	68.2	64.6 to 71.6	78.2	74.8 to 81.3
Main English-speaking country	10.0	9.0 to 11.1	10.5	9.1 to 12.2	9.2	7.5 to 11.3	9.2	7.5 to 11.2
Other	18.4	16.1 to 20.9	17.2	14.4 to 20.4	22.6	19.5 to 25.9	12.6	9.8 to 16.0
Remoteness								
Major city	73.1	70.7 to 75.3	67.2	63.9 to 70.4	76.7	74.5 to 78.6	69.4	66.3 to 72.3
Regional or remote area	26.9	24.7 to 29.3	32.8	29.6 to 36.1	23.3	21.4 to 25.5	30.6	27.7 to 33.7
Education								
Bachelor’s degree or higher	24.4	22.9 to 26.0	14.0	12.4 to 15.7	42.7	40.4 to 45.1	26.6	23.8 to 29.5
Adv. Dip., Diploma, Certificate or Year 12	46.6	45.1 to 48.1	42.8	40.2 to 45.4	47.5	45.5 to 49.4	52.4	49.7 to 55.0
Year 11 or below	29.0	27.4 to 30.7	43.2	40.5 to 46.0	9.8	8.8 to 10.9	21.1	19.0 to 23.3
Equivalised household income								
Middle or high income	80.0	78.0 to 81.8	62.0	58.9 to 65.0	78.6	76.6 to 80.5	58.3	55.1 to 61.5
Low income (lowest 40%)	20.0	18.2 to 22.0	38.0	35.0 to 41.1	21.4	19.5 to 23.4	41.7	38.5 to 44.9

* Only available for people with disability. Note that people may have multiple disabilities and belong to more than one disability group, thus the percentages do not tally to 100.

In both years, people with disability were older, more likely to be born in Australia and live in regional or remote areas compared with people without disability. A greater proportion of people with disability were male in 2003, while in 2023, a greater proportion were female. Although education levels increased for both groups, people with disability had a lower level of education and lower equivalised household income.

### Inequalities in employment status over time by disability

**Figure 1:**
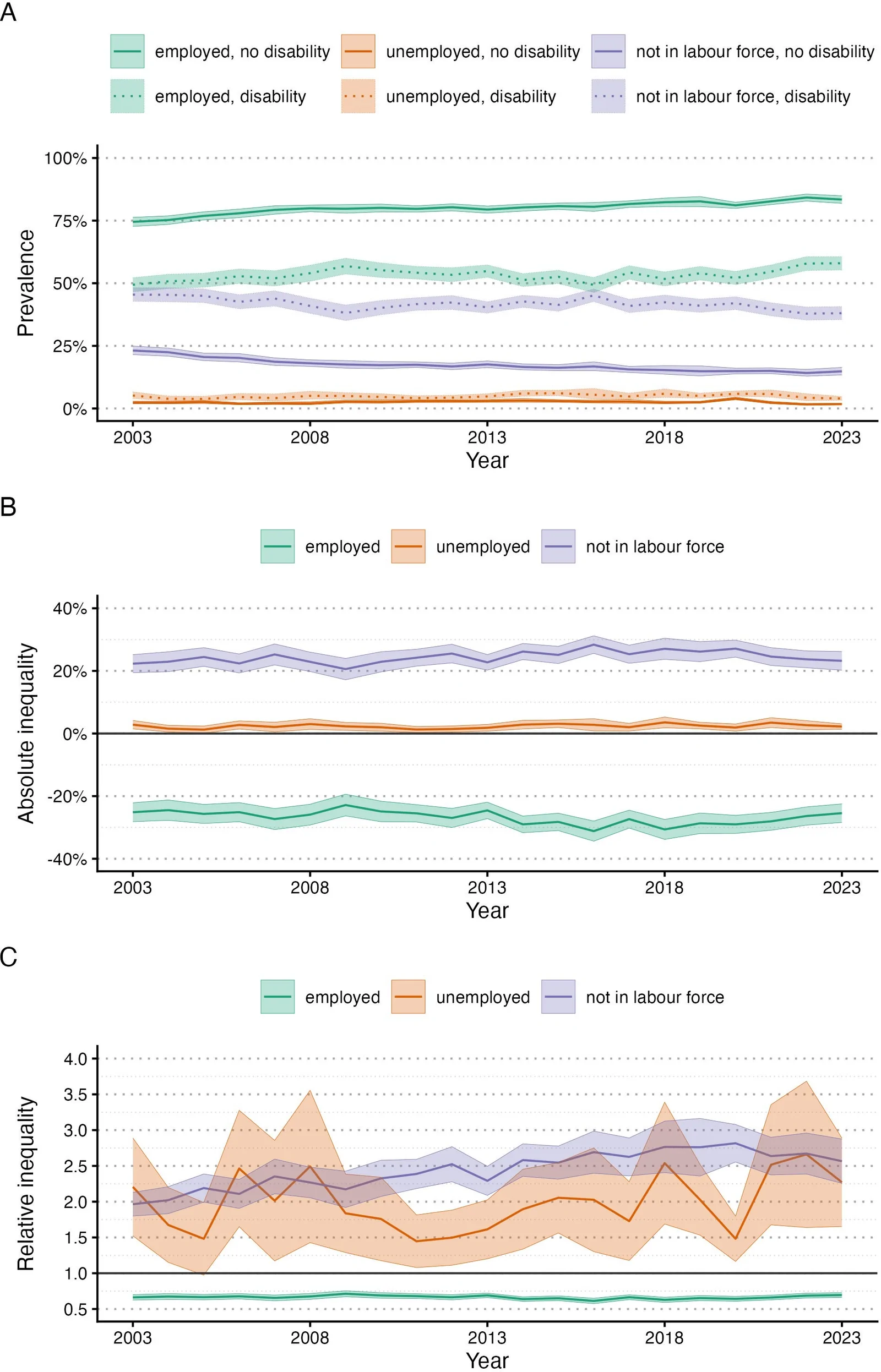
Employment status over time for Australians aged 20–64 years with and without disability, 2003–2023. (A) Age-standardised population-weighted prevalences; (B) absolute inequalities and (C) relative inequalities. Ribbons indicate 95% CIs for each estimate.

Figure 1 illustrates the prevalence, absolute and relative inequalities in employment, unemployment and NILF. The prevalence of employment increased between 2003 and 2023, from 49.4% (95% CI 46.6% to 52.2%) to 58.0% (95% CI 55.3% to 60.6%) for people with disability and from 74.5% (95% CI 72.7% to 76.3%) to 83.4% (95% CI 81.9% to 84.9%) for people without disability. Despite these improvements, neither absolute nor relative inequalities in employment changed over time, with overlapping 95% CIs over the 21 years. The largest inequalities in employment for people with disability were observed in 2016, with an absolute inequality in the prevalence of employment of 31.2 percentage points (lower) and a relative inequality of 0.6.

The prevalence of unemployment for people with and without disability, as well as absolute and relative inequalities, remained stable over time. In contrast, between 2003 and 2023, the prevalence of NILF declined for both groups, from 45.5% (95% CI 42.8% to 48.2%) to 38.0% (95% CI 35.5% to 40.7%) for people with disability and from 23.1% (95% CI 21.5% to 24.9%) to 14.8% (95% CI 13.4% to 16.4%) for people without disability. Despite this downward trend, while absolute inequalities in NILF remained stable over time, relative inequalities in NILF widened, increasing from 2.0 (95% CI 1.8 to 2.1) in 2003 to 2.6 times higher (95% CI 2.3 to 2.9) for people with disability relative to people without disability in 2023.

### Inequalities in employment status over time by disability and age group

**Figure 2:**
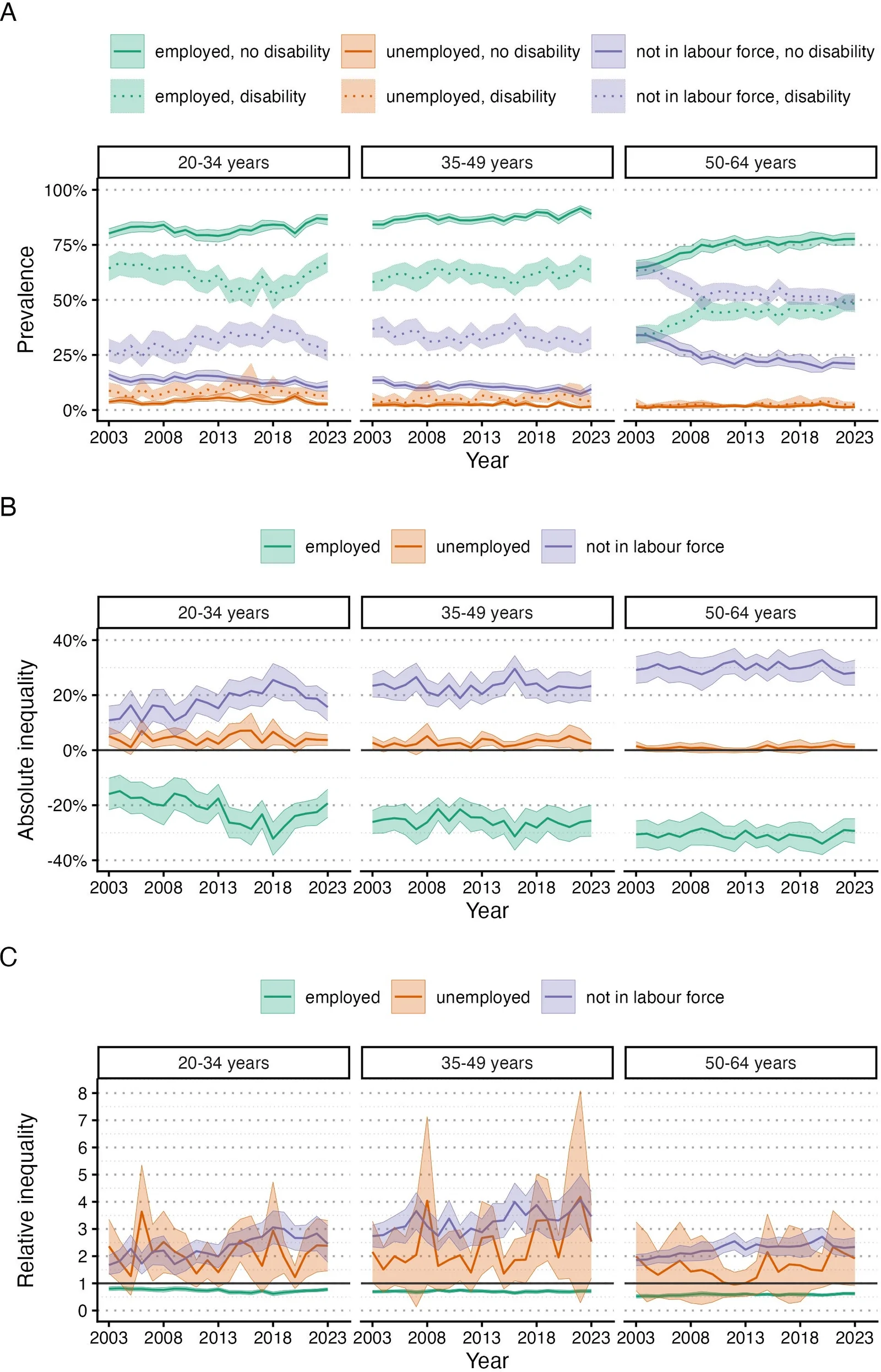
Employment status over time for Australians aged 20–64 years with and without disability, 2003–2023, stratified by age group. (A) Age-standardised population-weighted prevalences; (B) absolute inequalities and (C) relative inequalities. Ribbons indicate 95% CIs for each estimate.

Figure 2 shows that when stratified by age group, prevalence of employment was lower for both people with and without disability aged 50–64 compared with people aged 20 to 49 years. Absolute and relative inequalities increased with age, with the greatest inequalities in employment in 2023 observed among people with disability aged 50–64 years (absolute: 29.4 percentage points lower; relative: 0.7). When comparing trends over time, the prevalence of employment increased among people without disability across all age groups and people with disability aged 50–64 years. However, there was no evidence of change in absolute or relative inequalities in employment for any age group.

The prevalence of unemployment was lower for people with and without disability aged 50–64 years compared with younger groups. Both the absolute and relative inequalities in unemployment between people with and without disability persisted and remained stable among people aged 20 to 49 years and were only evident between 2021 and 2023 for the 50–64 years group. Considering NILF, although there was a greater proportion of people with and without disability aged 50–64 years being NILF and absolute inequalities increased with age, the relative inequality in 2023 was largest for the 35–49 year group (relative: 3.5). A declining trend in NILF prevalence was observed among people aged 50–64 years, regardless of disability. However, there was no evidence of change in either absolute or relative inequalities over time.

### Inequalities in employment status over time by disability and sex

**Figure 3:**
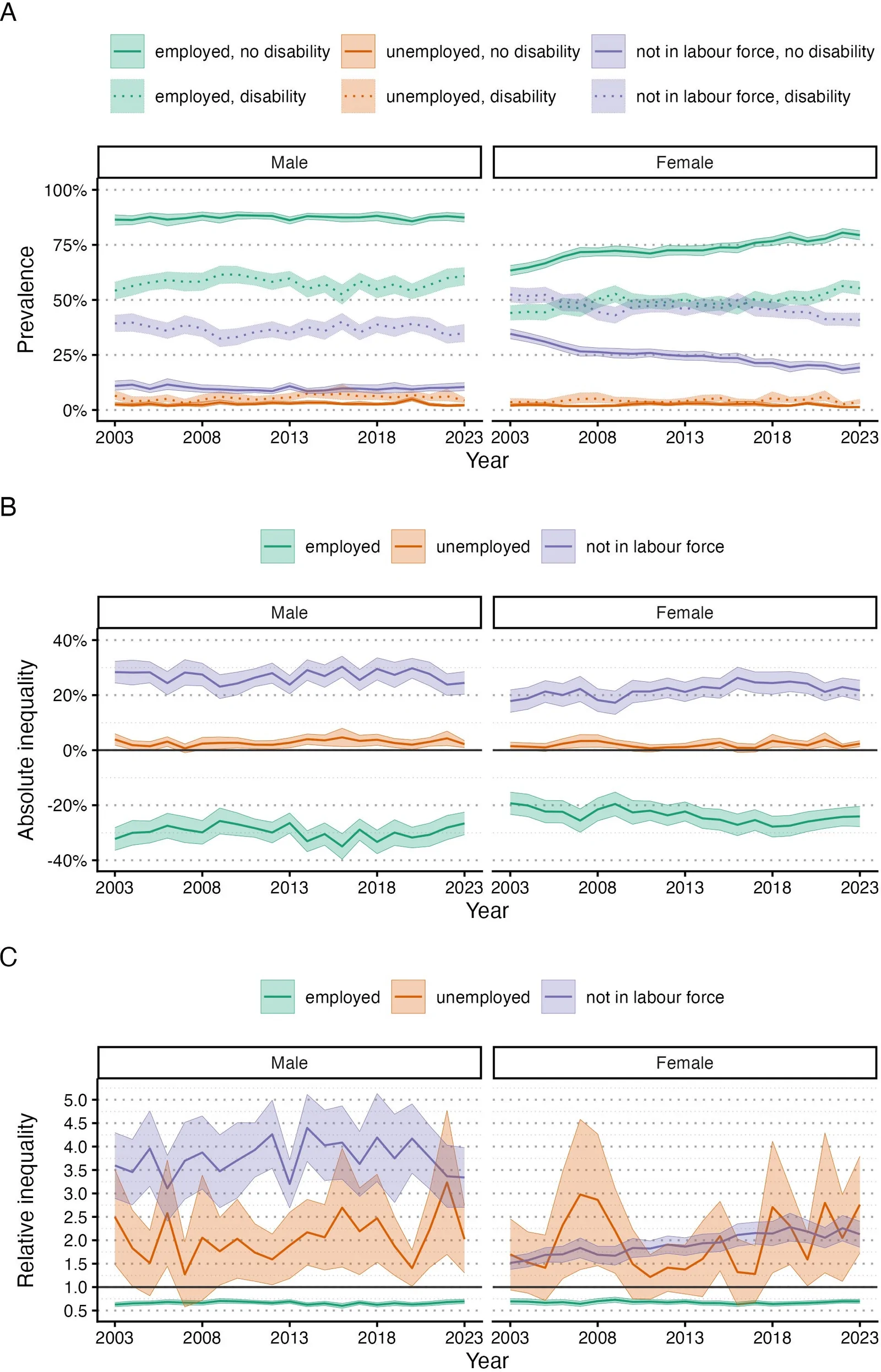
Employment status over time for Australians aged 20–64 years with and without disability, 2003–2023, stratified by sex. (A) Age-standardised population-weighted prevalences; (B) absolute inequalities and (C) relative inequalities. Ribbons indicate 95% CIs for each estimate.

Figure 3 shows that prevalence of employment was consistently lower for females than males without disability. However, for people with disability, this sex difference was only evident between 2003 and 2018. The prevalence of employment increased over time for females with and without disability but not for males. The absolute and relative inequalities in employment between people with and without disability were of similar magnitude for males and females, with no evidence of change from 2003 to 2023.

The prevalence of unemployment was similar between males and females with and without disability, and there was no difference in absolute and relative inequalities between males and females. There was no evidence of change in prevalence nor absolute or relative inequalities in unemployment for males and females over time. The prevalence of NILF was greater for females than males. However, the absolute and relative inequalities were larger for males than females. Although there was no change over time in prevalence, absolute or relative inequalities of NILF for males, for females there was a trend of decreasing prevalence of NILF, regardless of disability, and an increase in relative inequalities from 1.5 (95% CI 1.4 to 1.7) in 2003 to 2.1 (95% CI 1.9 to 2.4) in 2023.

### Inequalities in employment and NILF over time by disability group

**Figure 4:**
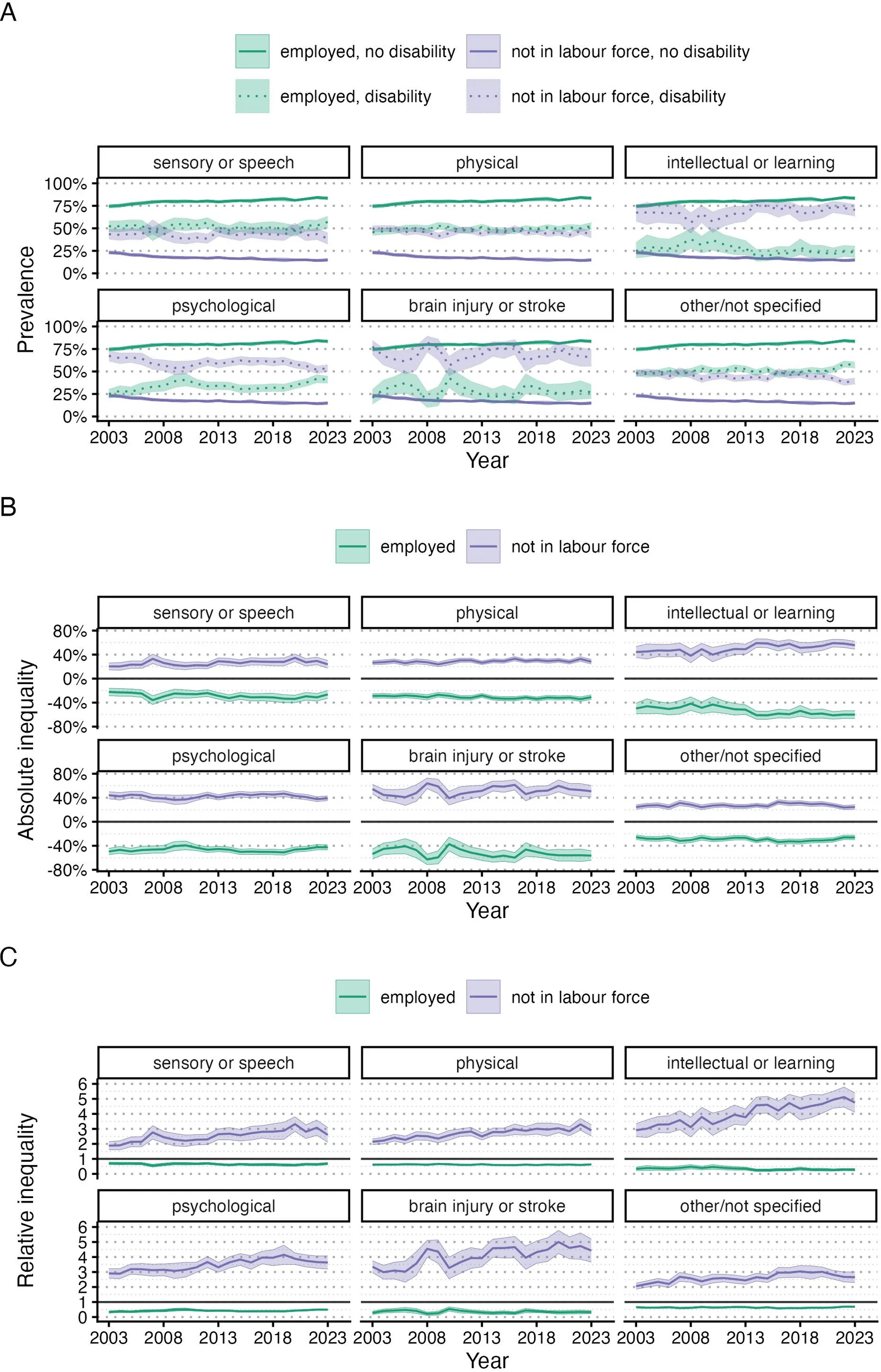
Employment and being NILF over time for Australians aged 20–64 years with and without disability, 2003–2023, stratified by disability group. (A) Age-standardised population-weighted prevalences; (B) absolute inequalities; and (C) relative inequalities. Ribbons indicate 95% CIs for each estimate.

Figure 4 shows that the prevalence of employment was lower for all disability groups than for people without disability, while the prevalence of NILF was higher for all disability groups; however, the extent of the differences varied between disability groups. In 2023, the largest absolute and relative inequalities in employment were observed for people with intellectual or learning disability (absolute: 60.1 percentage points lower; relative: 0.3), brain injury or stroke (absolute: 56.4 percentage points lower; relative: 0.3) and psychological disability (absolute: 42.6 percentage points lower; relative: 0.5). The magnitude of the relative inequalities in employment improved over time for people with psychological disability, increasing from 70% lower in 2003 to 50% lower in 2023. However, the relative and absolute inequalities in employment did not change for other disability groups.

The largest absolute and relative inequalities in being NILF in 2023 were also observed for people with intellectual or learning disability (absolute: 55.6 percentage points higher; relative: 4.8), brain injury or stroke (absolute: 50.9 percentage points higher; relative: 4.4) and psychological disability (absolute: 39.1 percentage points higher; relative: 3.6). Relative inequalities in being NILF increased between 2003 and 2023 for people with intellectual or learning disability (increase from 2.9 to 4.8), physical disability (increase from 2.1 to 2.9), and other/not specified disability (increase from 2.1 to 2.7), while remaining relatively stable for other disability groups.

## Discussion

This study found substantial and persistent inequalities in employment outcomes between people with and without disability across a 21 year period in Australia. From 2003 to 2023, people with disability consistently had lower prevalence of employment and higher prevalences of unemployment and NILF compared with people without disability, such that people with disability were 30–40 percentage points less likely to be employed, 1.5 to 2.7 times more likely to be unemployed, and 2.0 to 2.8 times more likely to be NILF than people without disability. There was no evidence of a change over time in the magnitude of either absolute or relative inequalities in employment or unemployment, and there was evidence of an increase in relative inequalities in NILF. Our findings are consistent with previous Australian^15 29^ and international studies^30^ that have reported employment inequalities for people with disability and add to previous findings by examining trends over time and demonstrating that the inequalities have not improved for more than two decades.

This study found persistent inequalities in employment for both males and females with disability compared with their peers without disability. Although males had higher prevalence of employment than females, the relative inequalities were similar. We observed that older adults were less likely to be employed and there were greater relative inequalities in employment between people with and without disability than for younger age groups. In addition, older people had consistently higher prevalence of NILF and there were lower relative inequalities in NILF between people with and without disability than for younger age groups. These findings suggest that early exit from the labour market may be common for people aged over 50 years, regardless of disability status. Poor physical health, including chronic conditions and functional limitations, is a key driver of early retirement,^31^ as declining work capacity may limit the ability to remain in paid employment. In addition, age-related discrimination and adverse working conditions, such as physically demanding jobs, particularly within Australia’s manufacturing-based economy,^7^ and poor psychosocial job quality,^32^ may further increase the likelihood of early labour force exit. Furthermore, some older adults may retire early voluntarily, including for leisure, family responsibilities, or perceptions of age-appropriate retirement norms.^31^

Although the type of disability can affect employment opportunities,^33^ we found that people from all disability groups experienced substantial and persistent employment inequalities compared with people without disability. The largest inequalities in employment were observed for people with intellectual or learning disability, brain injury or stroke and psychological disability. Although people with psychological disability experienced some improvement in relative inequalities in employment over time, the inequalities remained large. These findings are particularly important given the increase in prevalence of people with intellectual or learning disability and psychological disability in our sample. Our findings are consistent with a study using Australian national survey data that reported people with intellectual, psychological impairment or acquired brain injuries faced the greatest disadvantage in paid employment compared with other disability groups.^7^ Evidence also indicates that people with disability, particularly people with intellectual disability, were more likely to retire early or be forced into early retirement.^15^

### Public health and policy implications

The public health implications of our findings are considerable given that employment is a key social determinant of health. Empirical evidence shows that unemployment is detrimental to mental health for people with disability, with larger negative impacts compared with people without disability.^34^ Unemployment has been estimated to explain approximately 10% of mental health inequalities among working-aged adults who acquire disability^35^ and 20% among young people with disability.^29^ This is concerning given that the mental health of people with disability has worsened over the past decades in Australia, with widened mental health inequalities for people with intellectual or learning disability and brain injury or stroke.^36^

Employment has been a major priority in the UNCRPD and Australia’s disability policy frameworks over the past two decades, with substantial government investment directed towards disability employment services, supported employment and vocational training.^37^ Despite these investments, our findings show no evidence of improvement in employment inequalities between people with and without disability, suggesting that current policies and programmes have not effectively addressed barriers and improved opportunities to gain and maintain employment.

A major shift in Australian disability employment policy has been a transition from a focus on job-ready individuals towards more tailored support aligned with individuals’ goals, capacity and circumstances. For example, the NDIS Employment Strategy 2024–2026 and Employ My Ability place greater emphasis on flexible, effective and personalised employment supports, employer engagement, work aspirations and open and inclusive employment.^20 38 39^ Ongoing evaluation of recent reforms will be important to determine whether these policies lead to meaningful improvement in employment.

### Strengths and limitations

This study had several strengths. First, this is the first study to examine trends in employment inequalities between people with and without disability, and to further disaggregate these trends by age group, sex and disability groups. Second, we used a large, nationally representative sample of the Australian population over a 21 year period and calculated age-standardised population-weighted estimates to ensure appropriate representation of people with disability. Third, the HILDA Survey employs a consistent disability question aligned with the International Classification of Functioning, Disability and Health.^40^

However, there were also several limitations. First, the HILDA Survey is a household-based survey of people living in private dwellings and excludes people living in cared homes or correctional facilities who are more likely to have more severe disability. Nevertheless, only 3.3% of people with disability live in cared accommodation in Australia,^12^ thus the impact of this exclusion on representativeness may be limited. In addition, the HILDA Survey does not permit proxy reporting.^23^ As a result, people with more severe disability or communication impairments may have been underrepresented in this study, potentially leading to underestimation of employment inequalities. Second, the HILDA Survey has lower coverage for people living in regional, rural or remote areas, who may encounter additional structural barriers to labour market participation. However, we applied HILDA survey weights, which included a geographic component that would account for some of the differential coverage across regions. Third, we were unable to examine unemployment inequalities by disability group due to small sample sizes. Fourth, it was not possible to examine employment inequalities by severity of disability, as relevant measures in the HILDA Survey are only collected every four waves. Lastly, our study measured whether people were employed but did not consider other employment characteristics, such as work hours, employment types (eg, full time, part time, casual), voluntary and involuntary unemployment, underemployment, psychosocial job quality, income, occupation and mismatch between educational attainment and job level. Future research should investigate inequalities in these dimensions and factors contributing to employment inequalities between people with and without disability.

## Conclusion

Our findings demonstrate that people with disability in Australia have faced persistent and significant inequalities in employment outcomes over the past 21 years. These enduring inequalities have continued despite Australia’s commitments under the UNCRPD and extensive policy reforms aimed at improving employment outcomes for people with disability. Addressing these inequalities remains a critical public health priority, given that employment is beneficial for mental health, even more so for people with disability. Future research is needed to investigate inequalities in other employment characteristics and examine factors contributing to unemployment and persistent employment inequalities for people with disability. Strengthening policy monitoring and evaluation is essential to understand what policies work to make a difference to employment outcomes and to ensure that investments in disability employment initiatives effectively reduce inequalities.

## Data Availability

Data may be obtained from a third party and are not publicly available.
